# The role of necroptosis in adiposity-based chronic disease (ABCD): A review

**DOI:** 10.1097/MD.0000000000046901

**Published:** 2026-01-30

**Authors:** Yigao Wu, Juncai Bai, Guoqing Ma

**Affiliations:** a Department of Endocrinology, The Second Affiliated Hospital of Heilongjiang University of Traditional Chinese Medicine, Harbin, Heilongjiang, China; b Department of Cardiology, Zhengzhou University Affiliated Zhengzhou Central Hospital, Zhengzhou, Henan, China.

**Keywords:** adiposity-based chronic disease (ABCD), diabetes, diabetic complications, necroptosis, obesity, RIPK1/RIPK3/MLKL signaling pathway

## Abstract

Recently, as the obesity rate continues to rise and people’s understanding of obesity deepens, adiposity-based chronic disease has appeared in people’s view as a new obesity diagnostic term. As an endocrine, nutritional and metabolic disease, the mechanism of obesity is because energy intake exceeds energy consumption, resulting in excessive accumulation of body fat and abnormal weight, which can lead to a series of complications such as insulin resistance, diabetes mellitus, atherosclerosis (AS) and nonalcoholic fatty liver disease (NAFLD). In the last decade, a type of regulated and genetically controlled necrosis has emerged known as necroptosis. Necroptosis differs from non-immunogenic apoptosis in that it releases of cell contents and cytokines, which triggers an inflammatory response in adjacent tissues. Studies have shown that necroptosis is involved in the pathogenesis of obesity and its complications and that inhibition of necroptosis may reduce the progression of obesity-induced disease. In this paper, we discuss the role of necroptosis in adiposity-based chronic disease as well as the relationship between necroptosis and other cell death modes from the point of view of the mechanism of necroptosis.

## 1. Introduction

For a long time, it was generally believed that cell death was differentiated morphologically, with 2 modes of necrosis and apoptosis. Apoptosis is a caspase-dependent, non-immunogenic, active and controlled programmed cell death. In contrast, necrosis ruptures the plasma membrane, leading the contents to flow out, and is not controlled. In the past, the relationship between the 2 was generally considered antagonistic, however, our basic understanding of cell death has changed. It has been found that not all necrosis is passive, and cell death can be an active and regulated process. Necroptosis is a new mode of cell death. It is a cellular response to environmental stresses and can be caused by chemical and mechanical injury, inflammation and so forth. It is similar to apoptosis in that it strictly follows the intracellular signaling pathway and has the same morphological changes as necrosis.^[1,2]^ Necroptosis triggers an inflammatory response that exposes multiple organs to a necrotizing inflammatory environment, leading to dysfunction. There is a growing body of evidence that necroptosis is of great importance in a variety of inflammatory diseases.^[3]^

Here, we review the onset and development of necroptosis. Taking necroptosis as an entry point, we describe the current relevant findings that suggest a role for necroptosis in the process of developing obesity and its complications. Targeting necroptosis signaling pathways may provide ideas and directions for further research on obesity and its complications.

## 2. Necroptosis

### 2.1. Molecular mechanism of necroptosis

Necroptosis is a new non-caspase-dependent mode of programmed cell death mediated mainly by the activation of receptor interacting protein kinase 1/3 and mixed-lineage kinase (MLKL).^[4]^ Necroptosis has a unique signaling pathway that can be activated by death receptors or pathogen recognition receptors, etc.^[5,6]^ Recent studies have shown that necroptosis can also be triggered by extreme physical and chemical factors or severe pathological stimuli when severely damaged.^[7,8]^ Among them, the tumor necrosis factor receptor 1 (TNFR1)-mediated RIPK1-RIPK3-MLKL pathway is the most extensively researched pathway.^[9]^

RIPK1 and RIPK3 are key molecules in tumor necrosis factor-α (TNF-α)-mediated necroptosis pathway and TNF-α binds to one of 2 receptors, TNFR1 or tumor necrosis factor receptor 2. TNFR1 and intracellularly recruits RIPK1 and the death domain (DD)-containing adaptors, tumor necrosis factor receptor1 associated death domain protein, TNF receptor-associated factor 2, transforming growth factor-β-activated kinase-1, and cellular inhibitor of apoptosis proteins and other signaling molecules aggregated and formed complex I on the cell membrane. Both cellular inhibitors of apoptosis 1 and cellular inhibitors of apoptosis 2 are E3 ligases that cause polyubiquitination of proteins in complex I. RIPK1 serves as a platform for connecting signaling molecules, which in turn induces classical nuclear factor-κB (NF-κB) signaling. Subsequently, RIPK1 was freed to associate with caspase-8, RIPK3 and Fas-associated death domain (FADD) to form complex II.

**Figure 1.**
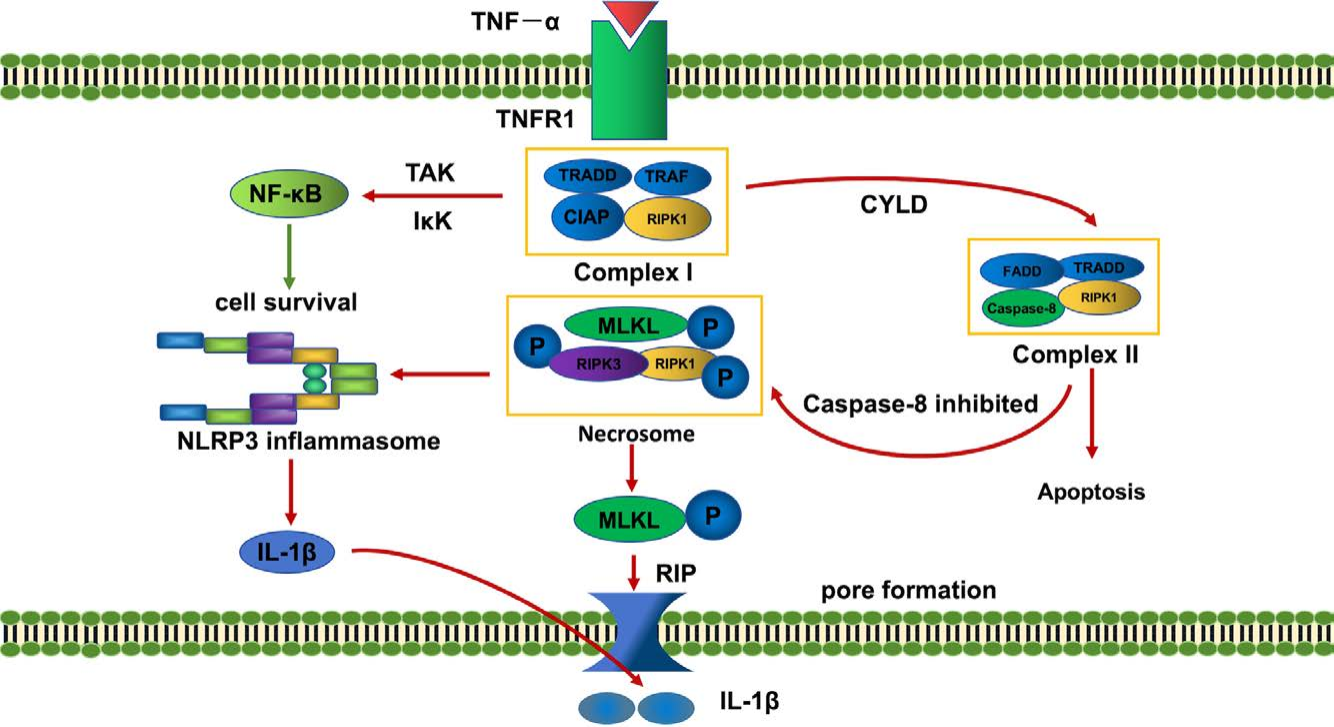
Mechanism of TNF-α-induced necroptosis. Upon TNF-α binding to TNFR1, the cell fate is determined by the activity of caspase-8. Active caspase-8 initiates apoptosis through the cleavage of downstream effectors. When caspase-8 is inhibited, the cell switches to necroptosis. This is executed through the phosphorylation of MLKL by the RIPK1/RIPK3 necrosome, leading to MLKL oligomerization, plasma membrane pore formation, and lytic cell death. cIAP = cellular inhibitor of apotosis protein, CYLD =* cylindromatosis.* RGB (color), FADD = Fas-associated death domain, IKK = IκB kinase, IL-1β = interleukin-1β, MLKL = mixed-lineage kinase domain-like, NF-κB = nuclear factor-κB, NLRP3 = nucleotide-binding domain, leucine-rich repeat, and pyrin domain-containing protein 3, RIPK1 = receptor-interacting protein kinase 1, RIPK3 = receptor-interacting protein kinase 3, TAK = transforming growth factor-activated kinase, TNFα = tumor necrosis factor alpha, TNFR1 = tumor necrosis factor receptor 1, TRADD = tumor necrosis factor receptor 1 associated death domain protein, TRAF = tumor necrosis factor receptor-associated factor.

Complex II comes in 2 forms: complex IIa and complex IIb, namely RIPK1 independent apoptosis and RIPK1 dependent apoptosis. The 2 can be distinguished by whether the activity of RIPK1 is required to induce apoptosis. TNF-α induces tumor necrosis factor receptor1 associated death domain protein, FADD, pro-caspase-8 and RIPK1 to form complex IIa, which promotes the activation of caspase-8, and the activated caspase-8 induces apoptosis through the activation of caspase-3. When caspase-8 is inhibited or its activity level is relatively low, RIPK1 and RIPK3 will not be cleaved, but will promote the deubiquitination of RIPK1, bind to RIPK3, FADD, and recruit MLKL through RIP homotypic interaction motif to form complex IIb, also known as necrosome. MLKL is activated by phosphorylation at T357 and S358. Oligomerized MLKL allows direct binding of lipids (myocardiolipin, phosphoinositol), forming permeable pores in the membrane with the polymerized MLKL, destroying the integrity of the cell membrane, and release cell contents, resulting in necroptosis^[10]^ (Fig. 1). It’s worth noting that in vitro, necroptosis requires the blockade of caspase-8 activity and apoptosis. However, several studies have found that the occur of necroptosis in vivo does not necessarily require caspase inhibition.^[11]^

### 2.2. Three key proteins of necroptosis

For the history of necroptosis, 3 key proteins are essential. In 2000, RIPK1 was found to participate in the regulation of necrosis signaling pathway.^[12]^ “Necroptosis” was named in 2005,^[13]^ and RIPK1 was shown to be the apical kinase in the pathway in 2008.^[14]^ In 2009, RIPK3 was identified as a kinase downstream of RIPK1.^[15,16]^ MLKL was later discovered as a terminal effector of necroptosis in 2012.^[17]^ In 2013, the initial crystal structures of MLKL and RIPK3 kinase’s structural domains were reported, along with the first MLKL knockout mice.^[18-20]^

RIPK1 is a key regulator of the innate immune signaling pathway and the first recognized signal molecule in the necroptosis pathway, including the N-terminal kinase domain, the C-terminal DD and the intermediate domain. RIPK1 is involved in TNFR1 signaling, activation of the NF-κB pathway, complex II formation, TOLL-like receptor signaling, and RIG-I-regulated antiviral responses, which is indispensable for cell survival, apoptosis and necrosis. However, RIPK1 is a multifunctional signaling kinase, and RIPK1 is solely validated by itself as its own substrate, whose autophosphorylation at Ser166 is considered to be a clue to the involvement of RIPK3 in the necrosome. Therefore, RIPK1 cannot be accurately used as a marker of necroptosis activation.^[21]^

RIPK3 is currently considered to be a central node in the necroptosis signaling pathway. RIPK3 is homologous to RIPK1, but misses the C-terminal DD compared to RIPK1.^[22]^ Self-oligomerization and autophosphorylation of RIPK3 are prerequisites for the execution of necroptosis and its activity determines the outcome of the cell towards necroptosis.^[16]^ Therefore, the detection of RIPK3 changes can reflect the occurrence and development of necroptosis.

Phosphorylated mixed-lineage kinase (p-MLKL) is the final actuator.^[23]^ An N-terminal 4-helix bundle domain and a C-terminal pseudokinase structural domain are linked by a d 2-helix linker termed the brace region, which makes up MLKL. When MLKL is phosphorylated, it forms p-MLKL with exposed 4-helix bundles. The p-MLKL forms a multimer and transfers to the plasma membrane of the cell, which causes rupture of the plasma membrane and facilitates necroptosis.^[24]^

### 2.3. Interplay between cell death pathways

Necroptosis, pyroptosis, apoptosis and ferroptosis are tightly linked and cross-regulate each other.^[25]^ While the exact mechanisms of their interactions remain unclear, there is value in exploring the similarities and differences between these various modes of cell death.

Apoptosis and necroptosis are both regulated by receptor interacting protein kinase (RIPK) and possess comparable signaling networks. Caspase-8, transforming growth factor activated kinase 1, and FADD are all involved in apoptosis and act as upstream mediators in regulating necroptosis.^[26-28]^ RIPK1 functions identically in exogenous and necroptosis, and inactivation of the kinase structural domain of RIPK1 can block both processes. However, their final steps are different, the kinase activity of MLKL or RIPK3 is necessary for necroptosis, but not for RIPK-dependent apoptosis. Cysteine aspartate proteases function as the executors of apoptosis. Importantly, in apoptotic cells, the plasma membrane remains intact, avoiding the release of cellular contents. Necroptosis, on the other hand, triggers an inflammatory response in adjacent tissues, through the release of cellular contents and cytokines. Caspase-8 functions as a molecular transducer, regulating apoptosis and necroptosis in a manner that hinges on the activity status and cell type involved. Studies have found that the expression of caspase-8 triggers the formation of ASC, the activation of caspase-1 and the release of interleukin-1β (IL-1β), and the activation process does not require the enzyme activity of caspase-8.^[29]^

Both pyroptosis and necroptosis are programmed cell death that strictly follows the regulation of intracellular genes. The endosomal sorting complexes required for transport-III complex acts as a common downstream mechanism to regulate necroptosis and pyroptosis sensitivity by ensuring membrane integrity through the release of p-MLKL^[18,30]^ or gasdermin d N-terminal-containing microvesicles^[31]^ from the cell surface. They are morphologically characterized by cytoplasmic osmotic swelling, mitochondrial dysfunction, formation of cell membrane pores, and release of intracellular contents causing an inflammatory response. However, pyroptosis is characterized by gradual flattening of cells, nuclear condensation, microstructures of pyroptotic vesicles, and gradual swelling of cells to the rupture of plasma membrane; whereas necroptosis is distinguished by cellular rounding and swelling, loss of nuclear chromatin, the formation of microstructures within necroptotic vesicles, and the abrupt rupture of the plasma membrane with a burst.^[32]^

A general balance exists among the various cell death pathways, and inhibition of 1 pathway may induce hypersensitivity to other pathways, for example, suppression of necroptosis may induces ferroptosis. Ferroptosis is a necrotic cell death caused by molecular dysfunction that prevents lipid peroxidation under healthy conditions. Ferroptosis diverges from necroptosis, pyroptosis, and apoptosis in numerous aspects. First of all, it is induced by non-classical signal transduction, for instance through DNA receptors or death receptors. Second, it is unclear how the plasma membrane ruptures during iron death. Finally, ferroptosis-induced cell death occurs in a manner that is non-cell-autonomous, termed synchronously regulated necrosis^[33,34]^ (Table 1).

**Table 1:** Comparison of cell necrosis, apoptosis, pyrolysis and necroptosis.

Items	Necrosis	Apoptosis	Necroptosis	Pyroptosis
Cellular morphology	Cell swelling	Cell shrinkage	Cell swelling	Cell swelling
Regulatory mechanism	Programmed	Unprogrammed	Programmed	Programmed
Cell membrance	Rupture	Intactness	Rupture	Intactness
Apoptotic bodies	No	Yes	No	No
Cellular content	Yes	No	Yes	Yes
Inflammation reaction	No	No	Yes	Yes
Inducing factors	Pathological stimulating factors	Death receptor signal/DNA damage etc	Death receptor signal/virus infection etc	Erastin/RSL3
Key molecules	None	Caspase-8/9/3/6/7	RIPK1/RIPK3/MLKL	Caspase-1/4/5/11/GSDMD
References	^[17]^	^[27]^	^[29]^	^[31]^

The table summarizes and contrasts the distinct morphological features, regulatory mechanisms, physiological consequences, inducing factors, and key executor molecules associated with the 4 major forms of cell death.

MLKL = mixed-lineage kinase domain-like, RIPK1 = receptor-interacting protein kinase 1, RIPK3 = receptor-interacting protein kinase 3.

## 3. An adiposity-based chronic disease (ABCD)

Obesity, as an endocrine, metabolic and nutritional disease, obesity is caused by energy intake exceeds energy consumption, resulting in excessive accumulation of body fat and abnormal weight, which can cause a variety of metabolic diseases. In recent years, as obesity rates continue to soar and awareness of its complications deepens, it has become imperative to assess its impact on various health conditions, the American Association of Clinical Endocrinologists and the American College of Endocrinology and the European Asylum Support Office have recommended the definition of adiposity-based chronic disease (ABCD) as a new term for the diagnosis of obesity. The aim is to move away from a single anthropometric measure, any body mass index (BMI), as a criterion for determining obesity, and to more accurately reflect an obesity-based pathophysiological process, with an emphasis on complication-centered management and staging strategies, effectively avoiding the stigmatization of affected individuals. The ABCD now features a novel disease classification scheme encompassing 4 distinct categories: A, B, C, and D. Group A codes depict the underlying factors of obesity, group B codes denote the BMI, group C codes delineate the complications linked to obesity, and group D codes specify the extent of those complications.^[35-37]^ The complications of obesity encompass diabetes, AS, cardiovascular disease, NAFLD and so on. In many cases, it is these complications, rather than obesity itself, that have a major impact on quality and expectancy of life, as well as longevity of the patients.

### 3.1. Role of necroptosis in diabetes and insulin resistance

Type 2 diabetes (T2D) is a speedily growing epidemic associated with changes in lifestyle and eating habits, as well as the prevalence of overweight and obesity.^[38]^ Excessive adipose tissue contributes to insulin resistance through an increase in the secretion of several adipocyte-derived proteins (adipocytokines).^[39,40]^

Glucose homeostasis in mice is associated with necroptosis proteins. Researchers have found up-regulation of MLKL, RIPK1 and RIPK3 expression in both adipose and liver tissues of obese mice.^[41,42]^ Gautheron et al^[41]^ found that RIPK3 inhibits inflammation, maintenance of tissue homeostasis and adipocyte apoptosis in white adipose tissue (WAT). Increased WAT inflammation and caspase-8-dependent apoptosis underlie glucose intolerance and are associated with impaired insulin signal in WAT, a result facilitated by inactivation of the RIPK3 gene. RIPK3 is also expressed excessively in visceral WAT of the obese individuals and is closely correlated with metabolic serum markers and BMI, much like in mice. Notably, in human patients, the overexpression of RIPK3 is closely related to the p-MLKL, a key target of RIPK3-mediated programmed necrosis. The occurrence and development of T2D and insulin resistance are inseparable from MLKL, a regulator of insulin sensitivity. In obese mice or wild-type (db/db, ob/ob, or diet-induced obesity), MLKL expression was upregulated in some obesity-correlated tissues and RIPK1 and RIPK3 were upregulated. Xu et al^[42]^ found that inhibition of the necroptosis protein MLKL could improve insulin resistance by modulating the phosphorylation of serine/threonine protein kinases induced by the insulin signaling molecule phosphatidylinositol-3,4,5-trisphosphate (PI-3,4,5-P3) in hepatocytes. The above results indicate that MLKL gene deficiency in mice significantly prohibits glucose intolerance and obesity-induced insulin resistance.

Several recent studies have suggested that pancreatic β-cell destruction may be associated with necroptosis.^[43,44]^ In addition, serum RIPK3 levels are elevated in patients with Corona Virus Disease 2019 (COVID-19).^[45]^

### 3.2. Role of necroptosis in DCM

The mechanism of diabetic cardiomyopathy (DCM) involves multiple regulatory elements, and there is an intricate and interdependent relationship between the factors.^[46]^ Myocardial cell death plays a major role in the initiation of DCM, and Cai et al^[47]^ reported that myocardial cell death was observed in both diabetic patients and animal models. Liu et al^[48]^ observed that diabetic rats with left ventricular remodeling and diastolic dysfunction were accompanied by necrostatin (Nec) and a significant increase in receptor-interacting protein 3 (RIP3) expression, suggesting that Nec is associated with myocardial injury in diabetic rats.

Recently, it was proved that the expression of key mediators of necroptosis (RIPK1, RIPK3, and MLKL) was increased in H9c2 cardiac cells subjected to high glucose (HG) stress, accompanied by down-regulation of cellular activation and expression of aldehyde dehydrogenase 2 (ALDH2), which has been associated with diabetic cardiac dysfunction.^[42,49]^ Suggested that necroptosis plays a key role in HG-induced cardiac inflammation and injury, which also confirms and expands the report of Liu et al^[48]^

Reactive oxygen species (ROS) accumulation and oxidative stress are among the major pathogenic mechanisms of diabetic complications.^[50]^ It was found that pretreatment of cardiomyocytes with *N*-acetyl-l-cysteine to inhibit ROS activity significantly antagonized the up-regulation of RIP3 by HG, indicating that one of the mechanisms by which oxidative stress damages the myocardium is by promoting the development of Nec. By detecting the influence of Nec-1 on HG-induced myocardial injury, it was found that Nec-1improved the secretion of IL-1β and TNF-α, significantly attenuated the increase of HG-induced RIPK3 expression, and inhibited HG-induced myocardial injury, which was manifested in the increase in cell viability, decreased ROS accumulation, decreased mitochondrial membrane potential dissipation, and decreased HG-induced IL-1β and TNF-α secretion.^[51,52]^ The above indicates that there may be a vicious cycle between necroptosis and ROS levels. It is worth noting that necroptosis is triggered by TNF-α.^[53]^ Therefore, a positive feedback loop between necroptosis and TNF-α might exist in HG-treated H9c2 cardiac cells. In order to confirm this hypothesis, further research is needed.

Hyperglycemia can damage cardiomyocytes by activating p38 mitogen-activated protein kinase (p38MAPK), NF-κB, leptin and other signal pathways.^[54,55]^ SB203580, an inhibitor of the p38MAPK pathway, was found to be able to counteract the HG-induced up-regulation of RIP3 expression. In a cardiomyocyte model of HG injury, HG activates the p38MAPK pathway and induces Nec. Nec can activate the p38MAPK pathway, and the activated p38MAPK pathway also promotes Nec. There is a positive interaction between the 2.^[56]^

MLKL is a vital enzyme downstream of RIPK3,^[57]^ but the changes in MLKL vary during different pathophysiological processes. One study found that in SIRT3-knockout (SIRT3-KO) diabetic mice, RIPK1, RIPK3 and cleaved caspase-3 protein expression expressed an upward trend and necroptosis was more intense, suggesting that SIRT3 deficiency exacerbates necroptosis in the myocardium. However, MLKL was not affected by diabetes induction and SIRT3KO, indicating that MLKL may not have any role in the development or advancement of DCM.^[58]^ Interestingly, in type 1 DCM, MLKL is essential for RIPK3-mediated necroptosis. It has been demonstrated that RIPK3 and MLKL may be activated concurrently within diabetic cardiac tissue and HG-induced cardiomyocytes; knocking down MLKL alleviates hyperglycemia-induced cardiomyocyte necrosis; furthermore, the activation of MLKL in cardiomyocytes and mouse hearts under diabetic conditions can be blocked by inhibiting RIPK3 via gene deletion or pharmacological inhibitors.^[59]^

Additional RIPK3 substrates, like Ca^2+^/calmodulin-dependent protein kinase II, may also participate in necroptosis induced by DCM. Inhibition of necroptosis has been found to improve myocardial mitochondrial ultrastructure by decreasing the level of oxidative phosphorylation of CaMK II.^[60,61]^ Necroptosis occurs in HG-induced H9c2 cardiomyocyte injury, suggesting that inhibition of necroptosis may have a cardioprotective effect.

### 3.3. Role of necroptosis in NAFLDl

NAFLD is defined as a disease in which there is an excess of liver fat due to nonalcoholic causes.^[62]^ There is growing evidence that programmed necroptosis is an etiological driver of apoptosis-associated liver disease and has a crucial role in NAFLD and nonalcoholic steatohepatitis (NASH).^[63]^

To date, multiple studies have used various NAFLD models for examining the role of necroptosis and have shown interesting and diverse results. RIPK1, RIPK3, and MLKL are pivotal signaling molecules responsible for triggering necroptosis in NAFLD models induced by high-fat (HF), choline-deficient HF, and methionine- and choline-deficient (MCD) diets. They also have a similar effect in db/db and ob/ob mice.^[64–66]^ Interestingly, the function of RIPK3 and MLKL in NAFLD varies depending on the disease model and condition. In RIPK3 (knockout, KO) rats, MCD diet-induced liver injury, inflammation, steatosis, and hepatic fibrosis were significantly attenuated compared to wild type. This indicates that the activation of RIPK3 may play a significant role in accelerating the course of liver disease.^[65,67]^ However, it has also been shown that RIPK3 deficiency exacerbates the expression of genes involved in the metabolism of glucose and fatty acids. The HF diet altered the programmed cell death balance towards augmented apoptosis, further aggravating fibrosis in RIPK3-KO mice. Compared to the wild-type mice, the RIPK3-KO mice demonstrate glucose intolerance, raised fasting glucose levels, and homeostatic model assessment of insulin resistance. In addition, RIPK3-KO mice showed elevated serum aminotransferases, hepatic steatosis, oxidative stress, and inflammation of the liver and adipose tissue. This shows that RIPK3 deficiency has a deleterious effect on HF-induced NAFLD.^[64]^ The varying impacts of RIPK3 deficiency in MCD and HF diets may stem from the differences in caspase-8 activity within these models and the different responses of adipocytes to the different dietary inducers.

The correlation between RIPK3 and MLKL expression was also observed in a human model of NASH. In 2015, Afonso et al^[67]^ observed a significant increase in hepatic RIPK3 levels among various patients with chronic liver disease, which correlated with the severity of steatohepatitis histology. p-MLKL is considered to be a protein kinase that performs necroptosis.^[17]^ Elevated expression of RIPK3 and MLKL has been discovered in human NASH biopsy samples. Moreover, the expression of RIPK3 and p-MLKL has been found to be elevated in the visceral adipose tissue from patients suffering from obesity and T2D. In addition, p-MLKL has a very similar granule staining pattern to that of RIP3 in NASH patients.^[65,67]^ However, research suggests that the impacts of removing RIPK3 and MLKL in NAFLD are inconsistent. While the deletion of RIPK3 has negative ramifications, the deficiency of MLKL appears to have a positive effect in NAFLD. TNF-α may mediate NAFLD-associated hepatocyte injury by triggering the necroptosis signal pathway in hepatocytes via stimulation of TNFR1. Serum TNF-α has been found to be elevated in NASH patients who are obese and also in the livers of NASH animals.^[67]^ Furthermore, the severity of liver disease interacts with TNF-α levels.^[68,69]^ The metabolic status of the liver is crucial in the development of NAFLD and impacts various programmed cell death pathways. This association ought to be considered while evaluating new molecular targets.

### 3.4. Role of necroptosis in DN

Diabetic nephropathy (DN) is a prevalent complication of diabetes mellitus and a leading cause of end-stage renal disease.^[70]^ As a necrotic-like response, necroptosis has emerged as a significant form of cell death in tubular injury.^[71,72]^

Renal macrophages have a close relationship with the level of renal damage, accumulation of renal interstitial matrix proteins, and interstitial fibrosis.^[40,73,74]^ Researchers have identified necroptotic apoptosis biomarkers, RIPK1, RIPK3 and MLKL, in renal tubular cells, demonstrating the involvement of necroptosis in the development of DN, and the same results were obtained in an in vitro experiment. In addition, the marker proteins’ levels were further enhanced, after co-culture with macrophages, indicating reveal that macrophages could be implicated in and support necroptosis of renal tubular cells.^[72,75]^

Podocyte damage and loss are associated with the onset and progression of DN.^[76]^ It has been found that in vitro HG treatment induces programmed necrosis and apoptosis of podocytes in renal tissues of patients with DN. Immunohistochemical examination of renal biopsies from patients with DN revealed increased renal expression of RIPK1, RIPK3 and MLKL in comparison with healthy renal tissue from renal tumor resection samples.^[75,77]^

It has been demonstrated that the progression of renal tubulointerstitial inflammation and fibrosis in streptozotocin-induced diabetic mice is reliant on RIPK1 and RIPK3, and that inhibition of the kinases reduces renal inflammatory cell infiltration as well as diabetes-induced collagen type I and III deposition, thus providing renal protection.^[78]^ Interestingly, HG status also induces activation of the M1 phenotype, production of the pro-inflammatory factor TNF-α, and activation of the podocyte ROS-p38MAPK signal pathway to induce podocyte apoptosis.^[79]^ Monocyte chemotactic protein-1 (MCP-1) is a chemokine that recruits macrophages to sites of inflammation. The macrophage M1 phenotype may induce podocyte injury by increasing the permeability of podocytes through MCP-1.^[80]^ It has also been shown that the MCP −1/CCR2 axis mediates foot cell apoptosis in diabetic conditions via the transforming growth factor-β (TGF-β) pathway.^[81]^ Macrophages play a crucial role in DN foot cell injury. Whereas, the exact mechanisms of necroptosis and DN remain complex, and the link between the 2 is not yet fully understood. Further research is required to investigate the mechanisms that would reveal fresh targets for the regulation of DN.

### 3.5. Role of necroptosis in DR

Diabetic retinopathy (DR) is a common microvascular complication of diabetes mellitus that often leads to vision loss in adults.^[82]^ It is widely accepted that the pathogenesis of DR is rooted in the thickening of the basement membrane of the retinal vasculature, which leads to pericyte loss and impaired function of the blood-retinal barrier.

Retinal ganglion cells (RGCs) make up the majority of retinal neural tissue, are the first neurons to differentiate from the retina, and are the initial cells that get affected in the early stages of DR, exhibiting the highest rate of apoptosis.^[83,84]^ After damage to the optic nerve, the extent of vision recovery is positively linked to the number of surviving RGCs. Retinal cells are affected by hyperglycemia, which increases peroxides and promotes apoptosis and viability of damaged cells, and necroptosis and retinal ischemia ensue.^[85]^ Recently, researchers found that the expression of RIPK1/RIPK3, the key protein in necroptosis induced by HG, was upregulated in vitro experiments, resulting in a decrease in Nissl bodies. The use of receptor-interacting protein 1 (RIP1) inhibitor (Nec-1) can significantly increase the amount of Nissl bodies in cells and inhibit necroptosis, which could effectively protect RGCs from d-glucose-induced necrosis.^[86]^ Previously, it has been demonstrated that prophylactic intravitreal injection of Nec-1 in rat eyes can protect inner retinal neurons and improve visual function.^[87]^

DR often causes ischemia of the retina, which gives rise to visual field loss and even blindness. Necroptosis has been shown to be involved in acute retinal ischemia-reperfusion and results in a range of inflammatory responses.^[83,88]^ In a retinal ischemia reperfusion injury (RIRI) model, the expressions of RIPK3 and RIPK1 were found to be significantly altered after RIRI injury, with mRNA levels starting to increase 1 day after RIRI, reaching a peak 2 days later, and then decreasing gradually. The change trend of RIP3 protein level was similar to that of mRNA. It is assumed that necroptosis is predominant in the incipient stage of RIRI, while apoptosis is predominant in the late stage of RIRI.^[89]^ It has been found that IR injury causes astrocyte activation and increased TNFα expression. In addition, IR-activated TNFα triggers the release of necroptotic apoptotic factors. The use of Nec-1 inhibited necroptosis and attenuated retinal cell damage.^[90]^ The above experimental results will enrich the theoretical basis for the involvement of necroptosis in DR damage and provide new possible targets for prevention and treatment.

### 3.6. Role of necroptosis in AS

AS is a chronic inflammatory disease of the vascular wall, driven by lipids and characterized by a non-adaptive process.^[91]^ There is growing evidence to suggest that necroptosis plays a crucial role in the pathophysiology of cardiac conditions, including vascular AS, myocardial infarction, ischemia-reperfusion injury, and cardiac remodeling.^[92–94]^

RIPK3 and MLKL were found to be detectable in advanced atherosclerotic plaques, and analysis of genetic data indicated greater expression of RIPK3 and MLKL mRNAs in atherosclerotic plaques than in normal arteries.^[95]^ Knockdown of RIPK3 in the apolipoprotein E (ApoE)−/− mouse background revealed a significant reduction in advanced AS lesions although there was no significant effect on early AS lesions, and similar results were observed in the Ldlr/mouse background.^[96]^ Another study demonstrated that decreasing necroptosis postponed mortality in mice and resulted in less severe harm to numerous tissues. The above suggests that intervention in the RIPK3 signal pathway may modulate the development of AS.^[97]^

Macrophage recruitment plays a significant role in the development of AS, and in advanced plaques, macrophage death is a key factor in the formation of necrotic cores. There are various forms of macrophage cell death within AS, necroptosis, apoptosis, autophagy-based death.^[98]^ AS-forming oxidized low-density lipoprotein (ox-LDL) processing of macrophages stimulates and promotes the transcription and phosphorylation of RIPK3 and MLKL, which is the mechanism by which necroptosis occurs in AS. Unlike traditional apoptosis, necroptosis releases inflammatory factors such as interleukin (IL) 6, IL-1α, MCP-1, etc, secondary to a series of inflammatory responses.^[97]^ Inflammatory factors can promote foam cell formation and plaque development, independent of hyperlipidemia. These factors have links to adverse cardiac outcomes in patients with AS. Although these markers have not been clinically validated, they may have deepened the understanding of the mechanisms of AS formation.^[99]^ A study in 2016 published p-MLKL as a marker of persistent necroptosis in human atherosclerotic plaques for the first time.^[95]^ In the mouse model of AS, knockdown of macrophage MLKL was found to reduce necroptosis and necrotic cores in plaques, suggesting that MLKL is an executioner of necroptosis and an important contributor to necrotic cores during AS formation.^[100]^

Endothelial dysfunction plays a crucial part in the occurrence and progression of AS, and an increasing number of studies indicate that necroptosis is linked to injury and death of endothelial cells in AS. ox-LDL responsively induces RIP1 and triggers NF-κB activation in treated human umbilical vein endothelial cells. Additionally, inhibition of RIP1 activity using Nec-1 improved the reduction of nitric oxide and formation of vascular adhesion molecules, in particular vascular cell adhesion molecule 1 and E-selectin, as well as the adhesion of immune cells to endothelial cells induced by ox-LDL. It is suggested that activation of RIP1 by ox-LDL may be a mechanism involved in endothelial damage.^[101]^ In another study, vascular peroxidase 1 (VPO1) was found to promote endothelial necroptosis and apoptosis hyperlipidemia by activating the β-catenin signal pathway, leading to endothelial dysfunction. VPO1 expression was increased in endothelial cells that were treated with ox-LDL, along with a reduction in glycogen synthase kinase-3 beta (GSK-3β) activity and p-β-catenin levels; meanwhile, stimulation of endothelial cells with hypochlorite instead of ox-LDL induced the same effect. These phenomena can be reversed using VPO1 siRNA or hypochlorite inhibitors.^[102]^

## 4. Conclusions

In summary, necroptosis serves as a pivotal autoregulated host cell defense mechanism, which also acts as a pathogen-suppressive defense mechanism. Thus far, several studies have shown that necroptosis can contribute to the genesis of obesity and associated chronic complications by instigating necroinflammation, particularly in diabetes mellitus, insulin resistance, retinal cells, cardiomyocytes, renal lamina propria, and liver injury. The curtailment of necroptosis noticeably ameliorates damaged tissue pathology. Furthermore, the collaborative utilization of various types of programmed cell death to elicit advantageous outcomes will be a crucial matter in regards to obesity and its associated complications.

## Author contributions

**Conceptualization:** Yigao Wu, Juncai Bai.

**Supervision:** Juncai Bai, Guoqing Ma.

**Writing – original draft:** Yigao Wu.

**Writing – review & editing:** Yigao Wu.
